# Role of Descending Serotonergic Fibers in the Development of Pathophysiology after Spinal Cord Injury (SCI): Contribution to Chronic Pain, Spasticity, and Autonomic Dysreflexia

**DOI:** 10.3390/biology11020234

**Published:** 2022-02-01

**Authors:** Gizelle N. K. Fauss, Kelsey E. Hudson, James W. Grau

**Affiliations:** Department of Psychological and Brain Sciences, Texas A&M University, College Station, TX 77843, USA; gizellefauss@gmail.com (G.N.K.F.); kelsey3hudson@tamu.edu (K.E.H.)

**Keywords:** spinal cord injury, monoamines, serotonin, GABA, neuromodulation, pain, autonomic dysreflexia, spasticity, ionic plasticity

## Abstract

**Simple Summary:**

Fiber pathways that descend from the brain to the spinal cord drive motor behavior and modulate incoming sensory signals and the capacity to change (plasticity). A subset of these fibers release the neurotransmitter serotonin (5-HT), which can affect spinal cord function in alternative ways depending upon the region innervated and the receptor type engaged. The present paper examines the dampening (inhibitory) effect of serotonin and how a disruption in this process contributes to pathophysiology after spinal cord injury (SCI). After briefly reviewing the underlying anatomy and receptor types, we discuss how damage to serotonergic fibers can enable a state of over-excitation that interferes with adaptive learning and contributes to the development of pain, spasticity, and the dysregulation of autonomic function (autonomic dysreflexia). Recent work has shown that these effects arise, in part, because there is a shift in how the neurotransmitter gamma-aminobutyric acid (GABA) affects neural transmission within the spinal cord, a modification that lessens its inhibitory effect. Clinical implications of these results are discussed.

**Abstract:**

As the nervous system develops, nerve fibers from the brain form descending tracts that regulate the execution of motor behavior within the spinal cord, incoming sensory signals, and capacity to change (plasticity). How these fibers affect function depends upon the transmitter released, the receptor system engaged, and the pattern of neural innervation. The current review focuses upon the neurotransmitter serotonin (5-HT) and its capacity to dampen (inhibit) neural excitation. A brief review of key anatomical details, receptor types, and pharmacology is provided. The paper then considers how damage to descending serotonergic fibers contributes to pathophysiology after spinal cord injury (SCI). The loss of serotonergic fibers removes an inhibitory brake that enables plasticity and neural excitation. In this state, noxious stimulation can induce a form of over-excitation that sensitizes pain (nociceptive) circuits, a modification that can contribute to the development of chronic pain. Over time, the loss of serotonergic fibers allows prolonged motor drive (spasticity) to develop and removes a regulatory brake on autonomic function, which enables bouts of unregulated sympathetic activity (autonomic dysreflexia). Recent research has shown that the loss of descending serotonergic activity is accompanied by a shift in how the neurotransmitter GABA affects neural activity, reducing its inhibitory effect. Treatments that target the loss of inhibition could have therapeutic benefit.

## 1. Introduction

In early development, neural excitability within the spinal cord is enabled, a process that fosters the emergence of neural circuits coupled by coherent patterns of activity [1]. Over time, operational modules form that help to organize motor behavior and regulate the transmission of sensory signals to the brain. As a stable network emerges, inhibitory processes develop that limit excitability and plastic potential. Part of this transformation is tied to a local alteration, attributed to a strengthening of the inhibitory potential of the neurotransmitter gamma-aminobutyric acid (GABA) [1]. Paralleling this change, serotonergic fibers from the brain innervate the spinal cord. These projections can have a neuromodulatory effect that can either facilitate or inhibit neural function depending upon the region/cellular systems innervated and the receptor systems engaged.

Damage to descending serotonergic fibers can impair motor performance and remove a homeostatic brake on neural activity that can fuel pathology after spinal cord injury (SCI). How serotonin (5-HT) modulates motor behavior (e.g., locomotion, respiration) and regeneration has been amply reviewed elsewhere [2,3,4,5,6,7,8,9]. Likewise, its role in regulating incoming pain (nociceptive) signals has been well covered [10,11,12]. The current review focuses on a different aspect of serotonergic function, how damage to these fiber pathways contributes to pathophysiology after SCI.

We begin with a brief review of the underlying anatomy, the receptor types engaged, how these affect neural functions, and the pharmacological tools used to study serotonergic systems. We then describe how a complete SCI (spinal cord transection) enables neural activity within the caudal tissue, a state that fosters plasticity. Attenuating inhibition, this places the spinal cord in a vulnerable state wherein strong noxious stimulation can sensitize neurons within the dorsal horn, a modification that interferes with adaptive learning, promotes cell loss when the spinal cord is bruised (contused), and can drive the development of chronic pain. The loss of serotonergic fibers has also been linked to the sustained motor activity (spasticity) and the dysregulation of autonomic function. The latter can allow nociceptive signals to trigger bouts of unregulated sympathetic activity (autonomic dysreflexia).

New work suggests that the loss of serotonergic activity enables over-excitation within the spinal cord because it transforms the action of GABA, recapitulating an earlier developmental state wherein its capacity to inhibit neural excitation is reduced. We discuss the neurobiological mechanisms that mediate these alterations and how treatments designed to quiet neural activity after SCI can bring therapeutic benefit.

## 2. Serotonin Function in the Uninjured Nervous System

### 2.1. Overview of Descending Pathways and Their Function

**Figure 1:**
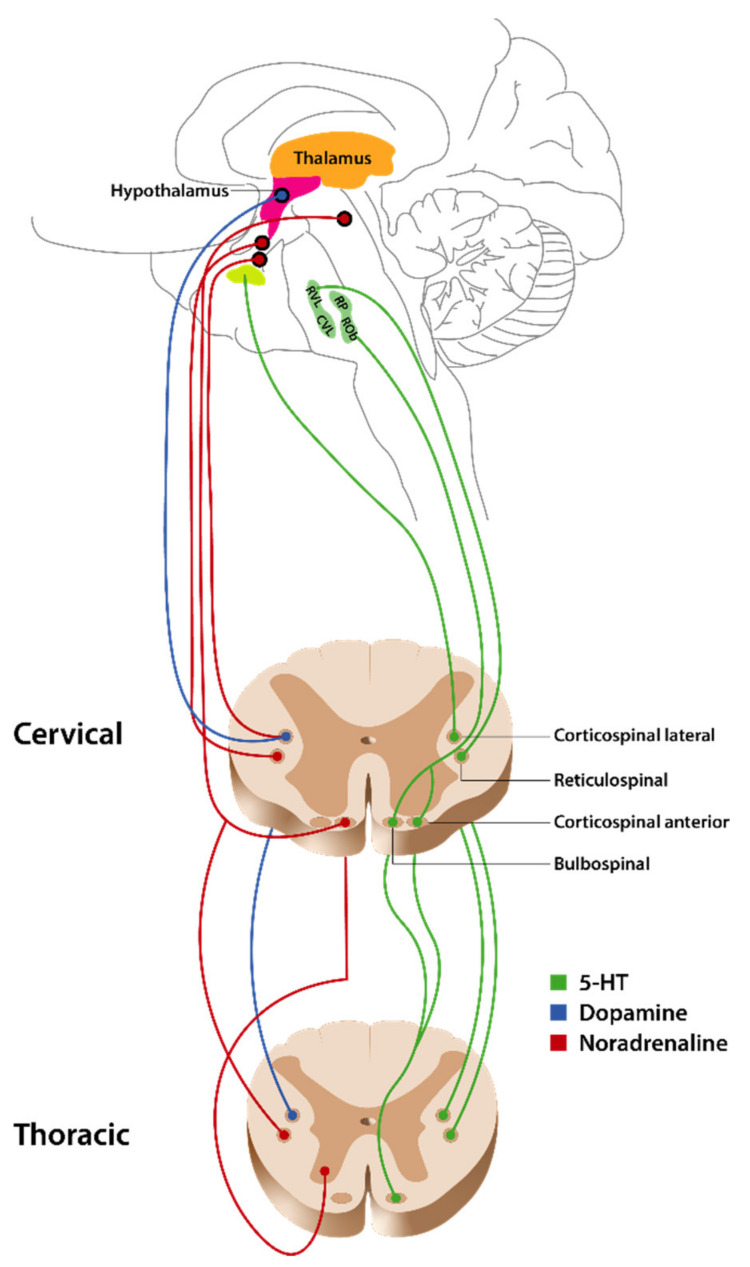
Serotonin (5-HT), noradrenaline, and dopamine projections to the spinal cord.

Traditionally, serotonergic systems within the rat brain were categorized into groups (B1–B9) by location, with B1 being the most caudal [13]. For the purposes of this review, we will focus on the medullary groups (B1–B3) of serotonergic fibers that descend into the spinal cord [14,15]. For further details on anatomy, see [16,17,18]. Groups B1 through B3 occupy regions of the raphe pallidus nucleus (B1), raphe obscurus nucleus (B2), and raphe magnus nucleus (B3). Serotonergic fibers project through the white matter of the spinal cord and terminate in three main regions: the dorsal horn, ventral horn, and intermediate zone (Figure 1). Fibers that terminate in the dorsal horn, a region that modulates nociception and sensory function, are mainly sourced from the raphe magnus nucleus and the adjacent reticular formation (the rostral ventrolateral medulla, group B3) [14,15,19]. These fibers travel through the dorsolateral fasciculus (DLF) and terminate primarily in laminae I and II of the dorsal horn. Motoneurons in the ventral horn (primary lamina IX) receive input from descending serotonergic fibers from the raphe obscurus and raphe pallidus (groups B1 and B2) [14]. In the thoracic cord, sympathetic neurons receive descending serotonergic inputs that are sourced from the ventrolateral medulla (group B3) [14].

#### 2.1.1. Regulation of Sensory Processes and Pain

The prominent sources of serotonergic efferents for nociception are the rostral ventral medulla (RVM) and the raphe magnus nucleus [14,15,20]. Within the dorsal horn, serotonergic fibers are most dense in the superficial laminae of the dorsal horn (laminae I and II) but the deeper laminae (IV–VI) also display serotonergic terminals [21,22]. Traditionally, modulation of nociception within the dorsal horn has been considered mainly inhibitory [23,24]. However, more recent data examining the effect of engaging alternative classes of 5-HT receptors suggest bidirectional modulation of nociception [16]. From this new perspective, it is not expected that engaging neurons within the raphe magnus or the rostral ventral medulla will necessarily induce antinociception. The outcome observed varies across stimulus parameters and both hyperalgesia and analgesia can be elicited by RVM stimulation [25,26,27,28,29]. Similarly, Ren and colleagues found that engaging vagal afferents projecting to the RVM could trigger facilitation or inhibition of nociception [30,31]. More recent reports have found specific “ON-cells” and “OFF-cells” in the RVM and raphe magnus that drive the facilitation and inhibition of pain, respectively [32,33].

Whether 5-HT has an antinociceptive or pronociceptive effect depends in large measure upon the receptor type engage (Table 1 and Table 2). 5-HT_1A/B/D_ and 5-HT_7_ are primarily antinociceptive while 5-HT_2_ and 5-HT_3_ are pronociceptive [34]. It is important to note that this grouping is general and that there is evidence for both anti- and pro-nociception for several of these receptors.

Work suggests that 5-HT_1A_ suppresses nociception by post-synaptically blocking dorsal horn neuronal activity [144,145,146,147,148,149]. There are also reports of 5-HT_1A_ receptor involvement in pronociception [41,42]. 5-HT_1B/D_ receptors, on the other hand, appear to only have an antinociceptive effect [40,57]. 5-HT_7_ receptors have multiple effects in modulating nociception depending on the physiological condition of the organism and the location of the receptors. In healthy rats, 5-HT_7_ receptor agonists exert a pronociceptive effect [121]. In neuropathic conditions however, 5-HT_7_ receptor agonists have an antinociceptive effect at the level of the spinal cord and pronociceptive effects at the periphery [120,121]. When agonists are administered systemically, however, the antinociceptive effect predominates over the pronociceptive effect in the periphery [120].

Pronociception is primarily mediated by 5-HT_2A/B_ and 5-HT_3_ receptors. Similar to 5-HT_1A_ receptors, there is evidence of both pronociception [40,73,74] and antinociception from 5-HT_2A_ receptors [71,72]. Unlike 5-HT_2A_ receptor, 5-HT_2B_ receptors appear to have only a pronociceptive effect [86,87,89]. While 5-HT_3_ receptors have also been characterized as pronociceptive [40,100,101], there are reports of antinociceptive actions [102,103].

#### 2.1.2. Regulation of Motor Behavior

It is well recognized that 5-HT also regulates locomotion and motor behavior [54,132,150,151,152,153,154,155,156,157,158]. For reviews, see [159,160]. For motor control, 5-HT pathways originate from the B1 and B2 regions of the medulla and project to the motoneurons and interneurons in laminae VII and VIII of the spinal cord [118,161,162,163]. The two main receptors that facilitate locomotion are 5-HT_2A_ [50,51,80,81,82] and 5-HT7 [51,81,82,124,164]. 5-HT_1A_ and 5-HT_2C_, however, are associated with inhibition of locomotor activity [50,51,94]. Importantly, 5-HT is also heavily involved in the neuromodulation of central pattern generator (CPG) activity [2,108,154]. Indeed, after SCI, CPG activity can be re-elicited by targeting 5-HT [54,84,165,166,167].

#### 2.1.3. Regulation of Autonomic Function

There are five major brain regions that modulate sympathetic function: the rostral ventromedial medulla, the rostral ventrolateral medulla (RVLM), the caudal raphe nucleus, the A5 region of the brainstem, and the periventricular nucleus of the hypothalamus [168,169,170,171]. Descending supraspinal vasomotor fibers that innervate sympathetic preganglionic neurons (SPNs) express numerous neurotransmitters including amino acids, catecholamines, and neuropeptides. Notably, serotonergic and noradrenergic inputs to SPNs are sourced from the caudal raphe nuclei and the A5 region of the RVLM, respectively [170]. These regions send projections to SPNs in the intermediolateral cell column throughout the T1-L2 segments of the spinal cord and regulate sympathetic outflow [172,173,174]. The RVLM is the primary source of input to supraspinal vasomotor pathways in the spinal cord that regulate cardiovascular function [175,176]. These fibers terminate in the dorsal and lateral funiculi in the spinal cord [177,178,179]. While sympathetic postganglionic fibers are driven by neurons from the T1-L2 region of the spinal cord innervate blood vessels throughout the body, the heart is innervated by SPNs innervated by neurons from the T1-T4 region of the spinal cord. Damage to this region can remove a regulatory brake on autonomic function, enabling the emergence of autonomic dysreflexia (AD) [18].

5-HT also affects parasympathetic function, an effect that is largely mediated by 5-HT_1A_ receptors. These signaling pathways have been implicated in parasympathetic control of respiration [180,181], heart rate [182,183,184,185,186], and micturition [44,45,46,47,48,49]. Other 5-HT receptors have been associated with micturition facilitation (5-HT_2A_, 5-HT_3_, 5-HT_4_, 5-HT_7_) [45,78,79,104,122], inhibition (5-HT_2C_) [78,79,95,96], and general function (5-HT_5_) [110].

### 2.2. Overview of How 5-HT Affects Neural Function within the Spinal Cord

The functional consequences of engaging alternative 5-HT receptors vary with the mechanism engaged and location within the spinal cord (Table 1). The 5-HT_1_ and 5-HT_5_ receptor families are negatively coupled to adenylyl cyclase through G_i/o_-proteins. Their activation leads to decreased production of cyclic adenosine monophosphate (cAMP), which ultimately leads to an inhibitory effect on neuronal firing [17,126,138]. The G_i/o_ receptor types are mainly found within the superficial dorsal horn of the spinal cord [35,55,56,60,61,110]. Other locations include the intermediate zone (5-HT_1B_) [35,55], ventral horn (5-HT_1D_) [62], and dorsal root ganglia (DRG) (5-HT_1F_) [66].

The 5-HT_2_ receptor family is positively coupled (via G_q_ proteins) to phospholipase C, which activates protein kinase C (PKC) and leads to increased accumulation of intracellular Ca^2+^. This class of receptors has an excitatory influence on neuronal activity. 5-HT_2_ receptors are primarily found within the spinal cord dorsal horn [69,86,91,92], with some expression in the ventral horn [69,70,88,91] and DRG (5-HT_2B_) [86,87] as well.

5-HT_4_, 5-HT_6_, and 5-HT_7_ receptors are positively coupled (via Gα_S_ proteins) to adenylyl cyclase, which, through protein kinase A, leads to an inactivation of K+ currents, exerting an excitatory effect on neuronal activity. The Gα_S_ receptor family is primarily distributed in the ventral horn of the spinal cord [106,114,118]. 5-HT_6_ and 5-HT_7_ can also be found in the dorsal horn [114,117] and 5-HT_6_ can also be found in the DRG [115].

Lastly, 5-HT_3_ receptors are exceptional to the 5-HT receptor family in that they are the only receptors that are ligand-gated and cation-permeable [17,187]. Upon activation, they enhance phospholipase C activity and facilitate neuronal excitability. 5-HT_3_ can be found throughout the spinal gray matter [91,98] and the DRG [99].

## 3. Impact of SCI on 5-HT Function

### 3.1. Impact of Injury on 5-HT Levels

5-HT response to SCI has been extensively studied (for review, see [83]). While the specific time course varies with species and SCI model, destruction of 5-HT fibers can induce an upregulation of 5-HT receptor expression that may last up to 3 weeks [92,156,188,189,190,191,192,193,194,195,196], with some reporting an extended effect sustained for 6 weeks [197] to 8 weeks [36]. Levels usually return to normal within 60 days [189,193] or earlier [198]. Higher levels have been reported within a few hours of injury [156,196,199] and after 24 h [192]. The upregulation of 5-HT, or its major metabolite (5-HT_1AA_), is associated with edema, increased vascular permeability, and decreased spinal cord blood flow [199,200].

After the initial increase, 5-HT levels decline [201,202]. Faden et al. examined 5-HT fiber immunoreactivity after a moderate to severe injury in rats [203]. After a severe injury, there was a near complete loss of 5-HT immunoreactivity within the lumbar spinal cord two weeks after injury, which was associated with severe spastic paraparesis (a decline in the capacity to move the hind legs accompanied by increased muscle tone and stiffness). In the moderately injured animals, they found a complete loss of staining in the dorsal horn and reduced staining in the ventral horn. In line with this less severe decrease of immunoreactivity, the rats showed moderate, spastic paraparesis. They concluded that loss of 5-HT fibers correlated with the severity of the SCI. Not surprisingly, motor scores were significantly correlated with changes in 5-HT staining in the ventral horn but not in the dorsal horn. This was attributed to the fact that the SCI significantly damaged the fibers in the dorsal region and that these fibers are linked to antinociception rather than motor function.

On the other hand, Saruhashi and colleagues reported that the recovery of serotonergic fibers correlates with gains in functional performance [204,205]. In a hemisection model [205], they found that 5-HT immunoreactive (5-HT+) fibers show increased expression in the ipsilateral cord after 4 weeks and that this predicts the time course and extent of locomotor recovery. The authors suggest the increased expression to be evidence of re-innervation. Similarly, in a later study [204], they found that an increase in 5-HT transporter terminal expression in the lumbosacral ventral horn also significantly correlates with locomotor recovery. Hashimoto, in 1991, found that 5-HT and norepinephrine (NE) are significantly correlated with neurologic score 14 days post-injury and thus suggest that they both participate in functional recovery [206]. Due to the disparate activity of 5-HT within the cord at different phases of injury, it is possible that 5-HT neurons have distinct roles in the progression of neural damage in the immediate phases of injury and in the recovery of function in the chronic phase of injury.

### 3.2. Acute Effects of Impaired Serotonergic Activity

#### 3.2.1. Descending Serotonergic Fibers Can Quell Nociceptive Sensitization

Damage to descending serotonergic fiber tracts will reduce 5-HT release independent of variation in presynaptic transmitter levels. The acute effect of damage to descending pathways on neural function within the lumbosacral spinal cord has been studied using a full thoracic transection, providing evidence that 5-HT release maintains a brake on neural activity within the dorsal horn that counters the development of over-excitation.

Work in this area was fueled by studies examining the effect of driving nociceptive input to the lumbosacral spinal cord after brain function was disrupted (e.g., by decerebration) or communication with the brain was blocked (by means of a rostral thoracic transection). Under these conditions, electrical stimulation of the sciatic nerve at an intensity that engages myelinated (delta) and unmyelinated (c) nociceptive fibers can induce a state of over-excitation within the lumbosacral dorsal horn [207]. Peripheral application of a chemical irritant (e.g., formalin) has a similar effect [208]. This phenomenon is often studied using the irritant capsaicin, which engages nociceptive fibers that express the transient receptor vanilloid 1 (TRPV1) receptor [209]. Treatment with capsaicin induces a lasting increase in neural excitability within the dorsal horn [210,211,212], a form of central sensitization [211]. At a cellular level, central sensitization within the spinal cord is correlated with increased expression of the immediate early proto-oncogene c-fos and the phosphorylation of the protein extracellular-signal-regulated kinase (pERK) [213]. At a behavioral level, nociceptive sensitization can transform how animals respond to light touch, leading to a withdrawal response when mechanical receptors are stimulated using calibrated (von Frey) filaments [214]. This alteration is of particular interest because it parallels the development of pain to touch (allodynia), a feature of neuropathic pain.

Interest in nociceptive sensitization was fueled by the observation that exposure to noxious stimulation can have a lasting effect, suggesting it may contribute to the maintenance of chronic pain [215]. Further work revealed that this memory-like effect depended upon signal pathways implicated in brain-dependent learning and memory, such as the N-methyl-D-aspartate (NMDA) receptor (NMDAR), calcium/calmodulin-dependent protein kinase II (CaMKII), and the trafficking/activation (phosphorylation) of α-amino-3-hydroxy-5-methyl-4-isoxazolepropionic acid (AMPA) receptors [210]. The link to brain-dependent learning and memory was further supported by work demonstrating that nociceptive stimulation can induce a form of long-term potentiation (LTP) within the dorsal horn and that this effect too depends upon the NMDAR [215,216]. Interestingly, nociceptive stimulation induces long-term depression (LTD) rather than LTP if the spinal cord is intact [217], implying that descending fibers normally inhibit the development of LTP.

Additional work revealed that brain systems inhibit the development of LTP within the dorsal horn via serotonergic fibers that descend through the dorsolateral funiculus (DLF), which inhibit nociceptive activity by engaging 5-HT_1A_ receptors [148,207,218,219,220,221,222]. This inhibitory effect has been related to the downregulation of adenylate cyclase and enhanced flow of K^+^ out of the cell [223]. Engaging the 5-HT_1A_ receptor can also counter the development of spinally mediated LTP by depressing voltage-dependent Ca^2+^ channel activity, which attenuates postsynaptic Ca^2+^ influx [148].

Recent work has shown that descending serotonergic systems also inhibit the development of spinally mediated nociceptive sensitization in response to treatment with capsaicin. Supporting this, Huang et al. (2016) showed that both behavioral (enhanced mechanical reactivity) and cellular indices of sensitization are amplified when communication to the brain is cut by means of a rostral thoracic (T2) transection [224]. Here too, the quieting effect was linked to serotonergic fibers that descend thru the DLF [127]. Supporting this, rostral cuts limited to the DLF fostered the development of nociceptive sensitization within the lumbosacral spinal cord. In animals that had undergone a complete transection, intrathecal (i.t.) application of 5-HT_1A_ agonist (8-OH-DPAT) to the lumbosacral region countered the development of nociceptive sensitization. Conversely, i.t. application of a 5-HT_1A_ antagonist (WAY-100635) in intact animals allowed nociceptive sensitization to develop. Taken together, the results suggest that descending 5-HT fibers normally quell the development of nociceptive sensitization, suggesting that this phenomenon may play a limited role in the maintenance of chronic pain in the absence of injury and/or inflammation. The corollary to this is that nociceptive sensitization is especially relevant to the emergence of chronic pain after SCI.

#### 3.2.2. Only Uncontrollable Stimulation Induces Nociceptive Sensitization

Further work revealed that the development of nociceptive sensitization within the spinal cord is modulated by behavioral control [225]. Behavioral control was introduced by applying noxious stimulation to one hind leg (via electrodes implanted in the tibialis anterior muscle) whenever the limb was extended [226]. Under these conditions, animals soon learn to maintain the leg in a flexed position, which minimizes exposure to noxious stimulation, a form of learning known as instrumental conditioning [227]. Subsequent work revealed that this learning involved an intraspinal modification and the NMDAR [228,229].

To show that introducing behavioral control mattered, animals in a second group were experimentally coupled (yoked) to those with behavioral control (master) [226]. Each animal in the yoked condition received electrical stimulation (shock) at the same time, and for the same duration, as its master partner but independent of leg position. Yoked rats that received this uncontrollable stimulation did not exhibit an increase in flexion duration—they failed to learn. Furthermore, they failed to learn when subsequently tested with controllable stimulation applied to the opposite leg, implying that treatment with uncontrollable stimulation induces a kind of learning deficit. Subsequent work showed that exposure to just 6 min of intermittent electrical stimulation applied in an uncontrollable stimulation impairs learning for up to 48 h [230].

Further research suggested that uncontrollable stimulation interferes with learning because it induces a state of over-excitation within the spinal cord, a form of nociceptive sensitization that saturates NMDAR-mediated plasticity and thereby interferes with the capacity to modify selective behavioral responses. Supporting this hypothesis, exposure to uncontrollable, but not controllable, electrical stimulation enhances reactivity to mechanical stimulation [231,232]. Furthermore, treatments that induce central sensitization (e.g., application of the irritants formalin, carrageenan, capsaicin) impair adaptive learning [231,232,233]. This learning impairment has been linked to an upregulation of the pro-inflammatory cytokine tumor necrosis factor (TNF) and the trafficking of Ca^2+^ permeable AMPARs [234,235].

#### 3.2.3. Uncontrollable Stimulation Increases Tissue Loss and Impairs Recovery after a Contusion Injury

Because over-excitation after SCI can foster cell death [236], exposure to noxious stimulation could increase tissue loss (secondary injury). This is clinically important because many injuries are accompanied by additional tissue damage (polytrauma) and invasive surgery is often needed to relieve pressure at the site of injury. To explore these issues, rats received a bruising (contusion injury) to the lower thoracic spinal cord using a surgical impactor. Nociceptive fibers were engaged the next day by exposing animals to intermittent electrical stimulation to the tail or the irritant capsaicin applied to one hind paw. Both treatments impaired long-term behavioral recovery [237,238]. Importantly, noxious electrical stimulation only impaired behavioral recovery if given in an uncontrollable manner [237]; stimulation had no effect when animals had behavioral control. Further analyses revealed that noxious stimulation increased the area of tissue loss at the site of injury and that this effect was related to increased expression of TNF [237,239]. Noxious stimulation also engages interleukin-1 beta (IL-1ß), IL 18, and signals related to cell death (caspase 1, 3, and 8) [238,239].

While it is not known whether 5-HT can counter the acute adverse effect nociceptive stimulation has on recovery, there is evidence that targeting spinal 5-HT soon after injury can improve cell survival and reduce neural damage. Bharne et al. found that giving spinally injured mice a 5-HT antagonist (ritanersin) and an alpha-melanocyte stimulating hormone resulted in reduced demyelination, necrosis and cyst formation, and improved locomotor recovery [240]. Administration of the SSRI fluoxetine after SCI had a 5-HT-dependent modulatory effect on matrix metalloproteinase-9 (MMP-9) activation that lessened hemorrhage and the breakdown of the blood-spinal cord barrier (BSCB) [241]. The drug also improved long-term locomotor recovery. In a later publication [242], they found that fluoxetine alleviates cell death (oligodendrocyte cell death) by inhibiting microglial activation after SCI.

#### 3.2.4. Descending 5-HT Fibers Help Preserve the Capacity to Learn

Consistent with prior work, exposure to uncontrollable stimulation does not induce a spinally mediated learning impairment in the absence of injury [75]. In these experiments, rats were given uncontrollable intermittent electrical stimulation to the tail using a computer program that emulated the variable pattern produced by an animal that had behavioral control (master). As previously reported, Crown et al. showed that noxious stimulation induced a learning impairment in animals that had received a rostral (T2) transection [230]. However, when the same amount of stimulation was given prior to T2 transection, it had no effect on spinal function, implying that brain-dependent processes normally act to preserve the capacity for adaptive learning to enable selective modifications within the spinal network. Interestingly, inhibiting brain processes with the anesthetic pentobarbital had an effect analogous to spinal transection, allowing noxious stimulation to induce a learning impairment [243]. This observation is clinically important because it suggests that nociceptive signals during medical procedures under anesthesia may adversely affect spinal function.

Here too, brain systems counter the adverse effects of uncontrollable stimulation via serotonergic fibers that descend in the DLF to engage the 5-HT_1A_ receptor [75]. Supporting this, noxious stimulation induced a learning impairment in animals that had spinal injuries limited to the DLF. Replacing 5-HT via intrathecal (i.t.) application of a 5-HT_1A_ agonist (8-OH-DPAT) blocked the adverse effect uncontrollable stimulation has on learning in transected rats. Conversely, when uninjured rats were given a 5-HT_1A_ receptor antagonist (WAY 100635 i.t.) prior to spinal transection, uncontrollable stimulation induced a spinally mediated learning impairment [75]. The observation that engaging the 5-HT_1A_ receptor can have a protective effect is consistent with other work demonstrating that 8-OH-DPAT attenuates NMDA mediated overexcitation and cell death [244] and inhibits NMDA evoked intracellular signaling cascades in vitro [245].

Taken together, research suggests that engaging nociceptive fibers can induce a form of maladaptive plasticity after SCI that impairs long-term recovery [246,247]. These adverse effects are modulated by behavioral control and brain systems which exert a protective effect via descending serotonergic fibers [225].

#### 3.2.5. Behavioral Control and Brain-Derived Neurotrophic Factor (BDNF) Counter the Adverse Effects of Noxious Stimulation

Work by Crown et al. revealed that introducing behavioral control does more than counter the immediate (acute) effects of nociceptive stimulation; it engages a lasting protective effect that blocks the development of a learning impairment when animals are subsequently exposed to uncontrollable stimulation [248]. It also counters the development of capsaicin-induced nociceptive sensitization [232]. In addition, after a learning impairment has been induced by exposure to uncontrollable stimulation, it can be reversed by training animals with controllable stimulation (in the presence of a drug that temporarily blocks the expression of the learning deficit) [248].

These protective/restorative effects have been related to an upregulation of BDNF [249,250]. Supporting this, the beneficial effect of training is blocked by i.t. application of an immunoglobulin (IgG) for the tropomyosin receptor kinase B (TrkB) receptor (TrkB-IgG) that sequesters BDNF. Conversely, i.t. application of BDNF can substitute for behavioral training to prevent the induction and expression of the learning deficit [250]. Application of BDNF to the lumbosacral region also counters behavioral and cellular signs of capsaicin-induced nociceptive sensitization [251]. Likewise, exercise and locomotor training increase the expression of BDNF [252,253] which attenuate behavioral signs of chronic pain and spasticity after injury [254,255].

The results reviewed above suggest that BDNF has a restorative effect after SCI. These findings stand in contrast to other work that suggests BDNF contributes to the development of nociceptive sensitization in uninjured animals [256,257,258,259], an effect that has been related to inflammation and the activation of microglia [260]. The implication is that BDNF can have a bidirectional effect on neural excitability and plasticity. This may help maintain the balance between excitatory and inhibitory transmission, providing a kind of autoregulatory homeostasis [261,262,263]. This suggests that the effect of BDNF on spinal cord function depends upon factors related to neuronal injury and the overall state of neural excitation [264,265]. After injury, when the effect of nociceptive stimulation on neuronal excitation is amplified, BDNF has a quieting effect; in the absence of injury, activity-dependent BDNF release may promote nociceptive sensitization. It is not currently known whether this transformation of BDNF function is tied to factors related to the cellular context in which it acts (e.g., cellular signals tied to the general level of neural excitation (e.g., intracellular Ca^2+^ concentration)), or the presence/absence of descending fibers (and cellular signals engaged by these pathways). It has been suggested [266] that the switch in BDNF function is related to the expression of phospholipase C (PLC), an effector of 5-HT receptor activation; when PLC is present, BDNF promotes neural excitation, whereas in its absence BDNF has a quieting effect. Supporting this, Garraway et al. showed that BDNF promotes neural excitability in the presence of PLC [267]. The hypothesis also predicts that treatments that engage PLC should promote the development of over-excitation and impair adaptive learning in spinally transected animals. As predicted, treatment with an agonist dihydroxyphenylglcine (DHPG) for the metabotropic glutamate receptor 1 (mGluR1), which engages PLC, induces a learning impairment [268].

Taken together, the results suggest that uncontrollable and controllable stimulation have opposing effects on spinal cord plasticity; the former disables the capacity to learn whereas the latter has an enabling effect. Because these effects involve the modulation of plasticity (the plasticity of plasticity), they have been characterized as forms of metaplasticity [127,269]. The same could be suggested for descending serotonergic fibers that modulate plastic potential within the dorsal horn [148].

### 3.3. Long-Term Effects of Impaired Serotonergic Activity

#### 3.3.1. Damage to Serotonergic Pathways Promotes the Development of Neuropathic Pain

Further work has detailed the long-term consequences of damage to 5-HT fibers, which can dysregulate nociceptive transmission and foster the development of neuropathic pain. Work in SCI models of neuropathic pain has shown that serotonergic fibers respond differentially to injury depending on the location. Bruce et al., in 2002, examined serotonergic structural changes after a clip-compression injury (T12) in rats and found that tactile allodynia and hyperalgesia are associated with a reduction in serotonergic fibers caudal to the injury [270]. These findings are supported by other work that linked below-level allodynia to the loss of descending 5-HT fibers after SCI [271]. However, Bruce et al. also found an increase in immunoreactivity for serotonergic fibers rostral to the injury, raising the question of whether the development of pain is due to the increase in faciliatory fibers or the loss of inhibition. Continuing the work of Bruce et al., Oatway and collaborators have found that 5-HT_3_ receptors (5-HT_3_R), known for being pronociceptive in pain transmission, facilitate at-level mechanical allodynia after a thoracic SCI [272]. They attribute this effect to the increase in 5-HT fibers immediately rostral to their T13 compression injury. Furthermore, Chen et al. found that a sustained delivery of a 5-HT_3_ receptor antagonist, given intravenously over multiple days, reduces at- and below-level mechanical allodynia in rats with thoracic SCI [273].

Outside of SCI, injury to the peripheral nervous system (PNS) can lead to similar adverse effects within the dorsal horn. Sprouting of descending serotonergic fibers in the dorsal horn that modulate nociceptive transmission has been found in models of afferent nerve injury [35,274] and traumatic brain injury (TBI) [275].

#### 3.3.2. Damage to Serotonergic Pathways Fosters Spasticity

Spasticity after SCI is a product of overactive, unregulated motor neurons within the spinal cord that create muscle spasms. Weeks after disconnection from supraspinal input, spinal motoneurons compensate for the loss of 5-HT by transitioning into an excitable state, easily responsive to excitatory transmitters such as glutamate [276,277,278,279]. This leads to the activation of 5-HT receptors that facilitate sustained firing of voltage-gated persistent Ca^2+^ and Na^+^ currents (also called persistent inward currents, PICs) and cause muscle contractions [280]. PICs and spasms are easily triggered by innocuous stimuli such as touch or muscle stretching [281]. Murray et al. [58,90,97] showed that this effect is due to the activity of 5-HT_2_ receptors. Supporting this, they found that tail spasms in rats after chronic transection injury are associated with constitutively active 5-HT_2_ receptors. Furthermore, administration of a 5-HT_2_ inverse agonist SB206553 (cyproheptadine) decreased the magnitude of the PICs and reduced the spasms in the rats. In the follow-up studies, they found that 5-HT_2B_ and 5-HT_2C_ receptors are responsible for the facilitation of motoneuron PICs [90]. Interestingly, they also demonstrated that 5-HT_1B/1F_ agonists can restore serotonergic inhibition of sensory transmission without affecting motoneuron function [58]. The authors showed that the pharmacologic control of 5-HT_2_ PICs is clinically relevant by administering the inverse agonist to spinally injured humans with evoked leg muscle spasms [97]. The drug significantly decreased muscle spasms. A subsequent study replicated the prior observation that cyproheptadine decreases CaPICs and showed that a serotonin reuptake inhibitor increased spastic muscle activity, further supporting the hyperactivity of 5-HT receptors [282]. The authors stress caution in choosing the dose of the drugs to preserve residual function of the motoneurons. When given at a high dose, cyproheptadine dramatically reduced weight support in rats with a staggered hemisection [97]. In addition, low doses of cyproheptadine have been shown to improve locomotor function in human SCI patients [283].

#### 3.3.3. Damage to Serotonergic Pathways Fosters Autonomic Function

Sympathetic dysfunction, in the form of blood pressure and cardiac impairment, is a prevalent comorbidity in SCI patients with high thoracic injuries. It often takes the form of a condition known as autonomic dysreflexia (AD), characterized by acute bouts of hypertension and bradycardia induced by innocuous or nociceptive stimuli below the injury (such as bladder or colorectal distension). It is well known that descending monoaminergic fibers are involved in spinal sympathetic regulation; spinal 5-HT_1A/2A_ receptors regulate blood pressure [284,285,286,287], activation of descending 5-HT axons produce elevations in arterial pressure [288], and adrenal receptors are involved in cardiac dysfunction induced by AD [289]. AD often occurs as a result of the loss of descending sympathetic fibers above the T6 region [18]. Loss of high thoracic supraspinal input can lead to the development of unmodulated sympathetic reflexes and decreased vasomotor tone that results in significant unregulated changes in blood pressure and heart rate that can be life threatening. Additionally, AD is associated with maladaptive fiber sprouting [290,291] and anatomic reorganization [292,293,294,295,296].

Loss of serotonergic fibers can foster the development of AD. In a rat model of severe SCI, it was found that a decline in 5-HT+ fibers located in the intermediolateral cell column of the spinal cord was associated with the severity of AD [297]. Intrathecal administration of a 5-HT_2A_ agonist restored resting mean arterial pressure (MAP) and blocked the colon distension-induced AD while a 5-HT_2A_ antagonist (ketanserin) had no effect on hypertension [297]. The serotonergic fibers were further characterized in a study in 2013 that examined axon regeneration using biotinylated dextran amine (BDA) injected into the rostral ventrolateral medulla to anterogradely trace the vasomotor pathways [179]. The authors observed localized labeling within the DLF throughout the cervical and thoracic spinal segments and, surprisingly, within the ventral white matter. A T4 hemisection that disrupted DLF fibers did not abolish the labeling or result in hemodynamic dysfunction. Only a complete bilateral transection injury that disrupted all supraspinal vasomotor pathways promoted the development of AD. In a subsequent study, the authors attempted to restore basal cardiovascular functions by injecting either brainstem-derived neural stem cells or spinal cord-derived neural stem cells into the T4 transection site [298]. While they found that both grafts mitigated AD, only the brainstem-derived cells displayed axonal growth and functional innervation. Additionally, graft-derived catecholaminergic and serotonergic neurons extended from the injury site and formed synaptic connections with the surrounding host tissue, suggesting that the regeneration of these fibers contributed to the cardiovascular functional recovery. Significant re-innervation was also observed when 5-HT+ neuron-enriched embryonic raphe nucleus-derived neural stem cells were grafted into the lesion site of T4 transected rats [299]. Functional innervation of serotonergic circuits regulating autonomic activity was associated with restored MAP and the alleviation of naturally occurring as well as artificially induced AD. These effects were mediated by the activity of the 5-HT_2A_ receptor, evidenced by the reversal of the grafting treatment with the 5-HT_2A_ antagonist, ketanserin. Lastly, in a study examining the effects of 5-HT_2A_ receptors and dopamine receptors in a rat model of AD, it was found that only 5-HT_2A_ receptor blockade restored hemodynamic parameters [300].

Recent studies have shown that AD after SCI is associated with cardiac dysfunction, in the form of unregulated heart rate and reduced contractility. A study in 2020 compared cardiovascular outcomes after a T2 transection or C6 transection in rats [301]. It was found that hemodynamic function and cardiac outcomes were different after 12 weeks based on the location of injury. The authors reported that C6-injured rats display hypertension and bradycardia while the T2 transected rats exhibit tachycardia. Relative to T2 transected rats, the C6 transected rats had reduced sympathetic tonus support to maintain arterial blood pressure. These results shine a light on the variability of AD symptoms found in human patients that differ in injury location and severity. In a study specifically examining cardiac function as a response to AD, spinally transected rats (T3) were given repeated episodes of AD 2 weeks after injury to allow for normal secondary injury mechanisms to occur. Repetitively induced AD resulted in significant cardiac dysfunction evidenced by reduced basal contractility and the desensitized β-adrenergic receptors. The desensitization of the β-adrenergic receptors was surprising because other studies find that AD increases sensitivity [289]. The desensitization could be attributed to the repeated induction of AD as well the increased circulating catecholamines that occur during episodes of AD [302,303]. A similar pattern of cardiac dysfunction was observed in human SCI patients with recurring episodes of AD. With the increased risk of heart disease in SCI patients [304,305], these results could indicate a link between AD and the development of heart disease.

Spinal sympathetic adrenergic receptors are also involved in immunosuppression after high thoracic injury. Lucin and colleagues found an association between hypothalamic–pituitary–adrenal (HPA) axis and sympathetic nervous system dysfunction and the reduction of antibody synthesis and elevated splenocyte apoptosis [306]. These effects were mediated by NE acting on β2-adrenergic receptors and could be reversed with pharmacological blockade. Pharmacological blockade of both glucocorticoids (GC) and β2-adrenergic receptors has similar restorative effects and diminishes SCI-induced splenic lymphopenia and lymphocyte Bim levels (a pro-apoptotic protein) [307]. While the effects of the immunosuppression could only be found in the T3 transection model, the effects could be mimicked in the T9 contusion model with the application of a β2-adrenergic agonist. A study in 2013 associated the immunosuppressive effects of high thoracic SCI with AD [308]. They found episodes of AD increased as a function of time post-SCI and that experimental activation of AD exacerbated the immunosuppression and splenic atrophy. These effects were also alleviated by pharmacological inhibition of NE and GC receptors.

#### 3.3.4. SCI Facilitates Pulvinar Reorganization and Dysfunction

In this review, we have discussed how descending serotonergic circuits contribute to significant dysfunction after SCI. It is important to note however, that supraspinal circuits are also affected by SCI. Pulvinar dysfunction in the thalamus has been observed in patients with complete SCI [309]. The pulvinar nucleus is known to play an important role in contextual multi-sensory processing and gating [310,311,312]. Importantly, the excitability of pulvinar neurons is modulated by 5-HT [313]. Specifically, 5-HT was found to have a hyperpolarizing effect. After SCI, there is a reorganization of supraspinal circuits to compensate for the lack of proprioceptive feedback. A functional magnetic resonance imaging (fMRI) study found an increase in functional connectivity between the left pulvinar nucleus and regions of the left inferior frontal gyrus and left inferior parietal lobe in patients with complete SCI [309]. The authors suggest that the lack of afferents from lower motor centers could create an imbalance in sensory weighting, initiating a compensatory increase in cross-talk between multisensory association cortices through the pulvinar nucleus. Given this, the pulvinar could be a promising therapeutic target after SCI.

## 4. Descending Serotonergic Fibers Regulate the Inhibitory Effect of GABA

The findings reviewed above suggest that the loss of descending 5-HT fibers promotes the development of maladaptive plasticity by enabling a state of over-excitation that can fuel cell death and foster the development of spasticity, pain, and autonomic dysreflexia. This dampening effect has been traditionally linked to the direct consequences of engaging the 5-HT_1A_ receptor, which has an inhibitory effect on neural activity (see Section 2.2). More recent work has revealed a secondary consequence of interrupting 5-HT function, related to an alteration in how the neurotransmitter GABA affects neural excitability, which may help to explain its broad effect on spinal function. This new perspective is motivated by two observations that challenge traditional views of how GABA affects neural activity.

### 4.1. Pretreatment with a GABA-A Antagonist Blocks the Development of Nociceptive Sensitization after SCI

The standard view of GABA function presumes it inhibits neural activity, an effect that is primarily mediated by the activation of the GABA-A receptor, an ionotropic receptor that regulates the flow of the anion Cl^−^ across the cellular membrane [314]. In the adult central nervous system (CNS), neurons maintain a low intracellular concentration of Cl^−^ [1]. As a consequence, engaging the GABA-A receptor allows the anion to flow into the cell, which has a hyperpolarizing (inhibitory) effect.

Within the uninjured adult spinal cord, GABAergic interneurons regulate neural excitation and plastic potential, exerting an inhibitory effect that modulates motor excitability and quiets nociceptive activity [315]. Neural inhibition also limits plasticity, which helps preserve neural circuits over time. Given this characterization, it is naturally anticipated that local application of a GABA-A antagonist (e.g., bicuculline) would remove a brake on neural activity to promote motor output, the transmission of sensory signals to the brain, and plasticity. As predicted, i.t. bicuculline has a pronociceptive effect that enhances behavioral reactivity to noxious and non-noxious stimuli, inducing a state akin to nociceptive sensitization [316,317,318,319,320,321]. Conversely, administration of a GABA-A agonist (e.g., muscimol), or implanting cells that express GABA, attenuates neural excitation and behavioral reactivity [322,323,324].

Contrary to the standard view are data demonstrating that blocking the GABA-A receptor can sometimes have an antinociceptive effect that counters the development of nociceptive sensitization. For example, in diabetic rats, pretreatment with bicuculline attenuates the enhanced mechanical reactivity (allodynia) elicited by peripheral treatment with the irritant formalin [325]. Likewise, in rats that have undergone a thoracic (T2) transection, bicuculline reduces the nociceptive sensitization elicited by noxious electrical stimulation, capsaicin, and inflammation [224]. Here, blocking GABA does not remove a brake on neural excitation; instead, the opposite is observed, which suggests that after SCI engaging the GABA-A receptor can have a paradoxical effect that drives, rather than inhibits, neural sensitization.

### 4.2. Alterations in Intracellular Cl^−^ Impact How GABA Affects Neural Activity

A second observation that led to a paradigm shift stemmed from the recognition that there is a developmental shift in how GABA affects neural activity [326,327]. This alteration is driven by changes in the intracellular concentration of Cl^−^, which is controlled by two membrane bound proteins, the K^+^-Cl^−^ cotransporter (KCC2) and the Na^+^-K^+^- Cl^−^ cotransporter (NKCC1) that regulate the outward and inward flow of Cl^−^, respectively [326,328,329,330]. Because NKCC1 develops first, the inward flow of Cl^−^ is augmented early in development, which maintains a high intracellular concentration of the anion [331]. Under these conditions, engaging the GABA-A receptor allows Cl^−^ to flow out of the cell, which has a depolarizing (excitatory) effect [326,327]. Later in development, there is increased expression of KCC2, which lowers the intracellular concentration of Cl^−^. Now, engaging the GABA-A receptor allows Cl^−^ to enter the cell, producing a hyperpolarization that inhibits neural activity.

What transformed the view of GABA function is the recognition that intracellular Cl^−^ concentration is dynamically regulated in the adult CNS, a phenomenon known as ionic plasticity [332,333]. Evidence suggests that this change is largely due to a downregulation of KCC2, which attenuates the hyperpolarizing effect of engaging the GABA-A receptor. Indeed, if KCC2 is sufficiently downregulated, engaging the GABA-A receptor can have a depolarizing effect that drives neural activity and plasticity. Evidence suggests that a downregulation of KCC2 can foster the development of hippocampal LTP and contributes to a number of disease states, including epilepsy, addiction, and diabetes [1,325,326,334,335,336,337]. Of particular import in the present context, SCI has been shown to downregulate KCC2 caudal to the injury, a transformation that removes a brake on neural activity and plasticity [328,338,339,340]. While this may benefit recovery by enabling the adaptive re-wiring of neural circuits [127], it also removes a governor on neural excitation, which enables nociceptive sensitization and the development of neuropathic pain [315,328]. The downregulation of KCC2 also contributes to the emergence of prolonged muscle activity (spasticity) after injury and the weakening of inhibitory processes essential to rhythmic locomotion [339,341]. In addition, a GABA-dependent over-excitation impairs the adaptive re-wiring of neural circuits and the capacity to learn [342,343].

The discovery that a downregulation of KCC2 contributes to pain and spasticity after SCI has fueled the exploration of a new class of treatments, designed to re-establish GABAergic inhibition by promoting KCC2 activity (e.g., CLP-290, a KCC2 activator) or by reducing the inward flow of Cl^−^ with a NKCC1 inhibitor (e.g., bumetanide) [328,340]. Evidence suggests that these treatments can promote the adaptive re-wiring of spinal circuits, foster behavioral recovery, and attenuate the development of spasticity and pain [224,339,343]. The realization that SCI brings a shift in how GABA affects neural activity also helps to explain the paradoxical effect of blocking the GABA-A receptor after injury—because injury leads to a high concentration of intracellular Cl^−^, engaging the GABA-A receptor has a depolarizing effect. Under these conditions, pretreatment with the GABA-A antagonist would be expected to have an antinociceptive effect that counters the development of nociceptive sensitization [224].

### 4.3. Exercise and Training Re-Establish GABAergic Inhibition after Injury

We noted earlier that exercise and locomotor training can have a therapeutic influence after SCI, promoting motor behavior and attenuating the maintenance of chronic pain and spasticity [255,344]. New data have revealed that locomotor training has these effects, in part, because it helps to re-establish GABAergic inhibition by upregulating KCC2 [345]. Because GABAergic inhibition plays an essential role in the execution of rhythmic behavior [341], this fosters the recovery of stepping. In addition, it helps to explain why step training and exercise attenuate chronic pain and spasticity. The beneficial effect of locomotor training and exercise after SCI may be related to increased expression of BDNF, which upregulates KCC2 after injury [345]. Indeed, blocking BDNF counters the behavioral benefit of training and its effect on KCC2, suggesting that BDNF expression plays an essential role [344,346]. As noted earlier, BDNF also attenuates nociceptive sensitization after SCI and this effect too has been related to an upregulation of KCC2 [251].

We discussed above how BDNF can have opposing effects on nociceptive processing, countering the development of sensitization after SCI but generally promoting neural excitability in the absence of injury [256,257,258]. These alternative effects may be explained by its opposing action on KCC2. After SCI, BDNF upregulates the expression of KCC2, which would counter the maintenance of pain and spasticity [254,339]. In the absence of injury, BDNF downregulates KCC2 within the spinal cord, which would fuel nociceptive sensitization and the development of neuropathic pain [258,260,347,348]. The key question then becomes why does BDNF have opposite effects on KCC2 in injured and uninjured animals? One suggestion is that this is determined by the signal pathways engaged [266,332,333]. BDNF binds to the TrkB receptor, which can activate both Shc (src homology 2 domain containing transforming protein) and PLC. How these pathways affect KCC2 depends upon PLC: If PLC is absent, KCC2 is upregulated; if PLC is engaged, KCC2 is downregulated. In line with this hypothesis, PLC is downregulated within the spinal cord after injury and upregulated by locomotor training [255,345]. Alternatively, the effect of BDNF on KCC2 may be modulated by the intracellular concentration of Ca^2+^ leading to a downregulation when the concentration is high and an upregulation when Ca^2+^ levels are low [326]. Supporting this, neural injury does not transform how BDNF acts if the depolarizing shift is blocked with bumetanide [349].

### 4.4. The Shift in GABA Function Is Tied to the Loss of Descending 5-HT Fibers

Recent data has linked the downregulation of KCC2 after SCI to the loss of serotonergic fibers that descend through the DLF [127]. Supporting this, lesions limited to this region can flip how bicuculline affects the development of nociceptive sensitization. In sham operated rats, the drug has a pronociceptive effect. After bilateral lesions of the DLF at T2, KCC2 is downregulated and bicuculline has an antinociceptive effect [127]. Likewise, in uninjured animals, pretreatment with a 5-HT_1A_ antagonist (i.t.) reverses the action of bicuculline, causing it to have an antinociceptive effect that counters the development of capsaicin-induced nociceptive sensitization. Conversely, after a complete SCI (T2 transection) i.t. administration of a 5-HT_1A_ agonist (8-OH-DPAT) upregulates KCC2 and re-establishes the pronociceptive effect of bicuculline.

A key unanswered question concerns the impact of these manipulations on the affective/motivational consequences of nociceptive stimulation. Does the loss of descending serotonergic fibers, and the consequent switch in GABA function, alter the sensory signal relayed to the brain? To explore this issue, we examined the effect of bicuculline treatment on capsaicin-induced pain in a place conditioning task, wherein animals experience different treatments prior to being placed in distinctive environments (contexts) [127]. Rats received bilateral cuts of the DLF at T2 or a sham surgery. Over the next two days, the key groups were treated with capsaicin before they were placed in each context. On one day, animals received bicuculline (i.t.) prior to capsaicin treatment; on the other, they received the drug vehicle before capsaicin. Prior work has established that animals exhibit a conditioned aversion to the context where they experience greater pain [350]. In the present case, the focal question concerns the effect of bicuculline treatment on capsaicin-induced pain. If GABA inhibits pain, blocking GABA should enhance the painfulness of capsaicin, inducing a stronger aversion to that context. To establish whether this occurred, animals were given a preference test where they were free to enter either context. As expected, sham operated rats showed an aversion to the context where they had received bicuculline before capsaicin, implying that blocking GABA-A receptors within the spinal cord enhanced pain. However, bicuculline had the opposite effect in DLF-lesioned rats. These animals preferred the context where they had received bicuculline prior to capsaicin treatment, implying that the GABA-A antagonist had an antinociceptive effect. The results reinforce the claim that, after SCI, the engagement of the GABA-A receptor by GABA promotes nociceptive activity and pain.

Other research has shown that a disruption in descending serotonergic fibers contributes to the downregulation of KCC2 that drives spasticity and motor impairments [339,341]. Here, however, 5-HT appears to act via the 5-HT_2_ receptor [351]. Pretreatment with a 5-HT_2_ agonist ((4-bromo-3,6-dimethoxy benzocyclobuten-1-yl)methylamine hydrobromide (TCB-2)) upregulated KCC2 after SCI and attenuated mechanical and thermal hyperalgesia. TCB-2 did not, however, attenuate the development of neuropathic pain induced by peripheral nerve injury in SCI rats [352]. Interestingly, treatment with TCB-2 also counters the stress-induced downregulation of KCC2 within the ventral tegmental area, a modification that contributes to alcohol self-administration [335,353].

Serotonergic innervation may also help to explain the transformation in BDNF function, which Rivera et al. linked to the expression of PLC—an effector of engaging 5-HT receptors [266,332]. In adult animals, descending 5-HT fibers would drive PLC signaling, which would cause BDNF to downregulate KCC2 and foster nociceptive sensitization. Damage to descending 5-HT fibers would reduce PLC activity and transform the action of BDNF, causing it to upregulate KCC2.

While the above may help to explain the change in BDNF function, some key questions remain unanswered: (1) How does the development of descending fibers upregulate KCC2?; and (2) Why does damage to these pathways have the opposite effect? Regarding the first question, prior work has shown that the shift in GABA function coincides with the innervation of descending fibers [354,355]. Furthermore, transecting the spinal cord at an early age (before fibers reach the caudal spinal cord) blocks the upregulation of KCC2. Finally, prolonged treatment with a 5-HT_2_ agonist (DOI) during the first postnatal week can substitute for the lost innervation in transected animals to re-establish GABAergic inhibition [351]. As to the second question, the reduction in KCC2 observed after SCI in adults could be tied to the inhibitory effect of descending fibers. In vitro, artificially driving neural activity leads to a downregulation of KCC2 [266,332]. Likewise, epileptic activity can drive KCC2 down. From this perspective, KCC2 is downregulated after SCI because damage to descending serotonergic fibers removes a source of tonic inhibition, resulting in a state of prolonged neural activity. Under these circumstances, removing a brake on neural activity is biologically efficient and could help neurons survive in the face of increased metabolic load [326,332]. Interestingly, the initiation of this process may depend upon BDNF; blocking BDNF before neural activity is increased, or the spinal cord is cut, counters the downregulation of KCC2 [266,339].

## 5. Role of Other Monoamines

Monoaminergic neuromodulation within the spinal cord includes not only serotonin, but NE and dopamine as well. While descending serotonergic fibers have been the main focus of this review, it is important to acknowledge the role of the other monoamines.

### 5.1. Noradrenergic Fiber Pathways

Noradrenergic projections to in the spinal cord (Figure 1) are primarily sourced from the C1 and C2 medullary nuclei, the A_5_ and A_6_ nuclei in the locus coeruleus, and the A_7_ pontine region [17]. The intermediolateral cell column and the ventral horn are recipients of noradrenergic input from the A5 and A6 regions, respectively, while the dorsal horn receives input predominately from the A_7_ region. There are three major classes of adrenoreceptors (Table 3 and Table 4). The α_1A/B/D_ are characterized as excitatory through their positive coupling to G_q/11_ proteins. The α_2A/B/C_ receptors are characterized as inhibitory via their inhibition of adenylyl cyclase through G_i/o_ proteins and their suppression of Ca^2+^ currents. Lastly, β-adrenoreceptors (β_1/2/3/4_) stimulate neuronal activity via Gs proteins.

Adrenoreceptors’ involvement in spinal cord neuromodulation is extensive. Traditionally, in conditions of early SCI, NE’s activities are known to be involved in hemorrhagic necrosis [386,387,388,389], blood pressure/blood flow [390,391,392], and motor function [387,393,394,395]. Nociception is regulated by α2-adrenergic receptors which inhibit activity of deep dorsal horn neurons [362,363], and in neuropathic pain models of SCI, catecholaminergic fibers have shown evidence of maladaptive plasticity and fiber sprouting after thoracic transection [396,397]. Lastly, spinal sympathetic β-adrenoreceptors have been shown to be involved in immunosuppression after high thoracic SCI [306,307], and these effects have also been linked to AD [308].

### 5.2. Dopaminergic Fiber Pathways

Descending dopaminergic projections (Figure 1) originate from the A11 region of the periventricular posterior hypothalamus [17]. These fibers can be detected in the intermediolateral cell column and the ventral horn, but they are primarily found in the dorsal horn and lamina X. Dopamine receptors are classified into two families, D_1_-like and D_2_-like (Table 5 and Table 6). D_1_-like receptors include D_1_ and D_5_ receptors and they have an excitatory action upon neural activation via G_q_ proteins through stimulation of adenylyl cyclase [17]. D_2_-like receptors include D_2_, D_3_, and D_4_ and they inhibit adenylyl cyclase through G_i/o_ proteins and thus suppress neural activity.

While research on the role of spinal cord dopamine is growing, there is relatively little known regarding its contribution to pathology after SCI [415,416,417]. There is evidence it is involved in pain modulation. Supporting this, systemic administration of D_2_ receptor agonists elicit antinociception while D_1_ receptor agonists elicit pronociception [17,403,410,411,412,413]. In an SCI model, targeting D_2_ receptors has been shown to alleviate pain-related behaviors and even improved secondary injury by reducing inflammation and MMP-9 expression [407]. Lastly, dopaminergic agonists administered to the preganglionic neurons within the intermediolateral cell column have been shown to elicit hypotension and bradycardia [400,418,419,420].

Recent work has found that dopamine receptors play an active role in micturition after SCI. In a male rat thoracic (T10) transection model, it was found that spinal D1 receptors tonically suppress tonic external urethral sphincter (EUS) activity to enable voiding while the activation of D2 receptors facilitates voiding [399]. Work in a complete transection female rat model showed similar results where pharmacologic activation of D1 receptors after SCI inhibits urine storage and enhances voiding by differentially modulating (EUS) tonic and bursting patterns, respectively [398]. Additionally, they found that pharmacologic activation of D2 receptors with quinpirole improves voiding by enhancing EUS bursting.

## 6. Conclusions

### 6.1. Summary

We have described how damage to descending serotonergic fibers can contribute to pathophysiology after SCI. These effects include an amplification of nociceptive signaling that fosters the development of acute nociceptive sensitization, impairs adaptive learning and locomotor recovery, and promotes the development of neuropathic pain, spasticity, and autonomic dysreflexia. In many cases, these adverse effects appear tied to a loss of activity at 5-HT_1A_ and 5-HT_2_ receptors. New research has shown that the loss of serotonergic activity downregulates the co-transporter KCC2 caudal to injury, bringing a reduction in the inhibitory effect of GABA. It was suggested that this modification may provide a cellular context that fosters pathophysiology, to augment the adverse effects of nociceptive input, impair locomotor function, and drive spasticity. Treatments that bolster 5-HT function after injury may bring benefit by restoring GABAergic inhibition. Likewise, the pathophysiological consequences of damage to serotonergic fibers may be lessened by treatments that target ionic plasticity.

### 6.2. Limitations and Issues for Future Research

We described above how noxious stimulation can induce a state of over-excitation in the spinal cord and undermine recovery after injury [231,237]. We have also shown that pain input after injury engages pro-inflammatory cytokines and signals related to cell death [238]. More recently we discovered that nociceptive stimulation after SCI promotes hemorrhage at the site of injury [421]. Because blood borne contents are neurotoxic [422], the infiltration of blood would expand the area of tissue loss (secondary injury). Our review of serotonergic regulation of spinal systems has emphasized how these fiber tracts can quell over-excitation and thereby have a protective effect. Given these observations, we naturally hypothesized that noxious stimulation would lead to greater tissue loss and hemorrhage after a contusion injury if communication with the brain was cut. We found exactly the opposite—that disrupting communication with the brain by means of a surgical or pharmacological transection at T2 blocks nociception-induced hemorrhage in rats that had a lower thoracic contusion injury [423,424]. A T2 transection also blocked the activation of pro-inflammatory cytokines, and signals indicative of cell death, at the site of injury. Furthermore, a pharmacological transection at T2 blocked the adverse effect nociceptive stimulation has on long-term recovery [423]. Because a rostral transection is sufficient to downregulate KCC2 in the caudal tissue [224], these findings imply that the shift in GABA function does not, by itself, enable nociception-induced hemorrhage after injury [425]. The implication is that an additional, brain-dependent, process is engaged that plays an essential role in driving pain-induced tissue loss after injury. We have hypothesized that this adverse effect may be linked to a nociception-induced surge in blood pressure [426]. From this perspective, local alterations may enable nociceptive sensitization (setting the stage for chronic pain) and place the tissue in a vulnerable state (e.g., by weakening the blood spinal cord barrier). A surge in blood pressure/flow could then lead to hemorrhage, increasing inflammation and cell death at the site of injury.

A related issue that requires additional research concerns the dissociation of the time-course of injury-induced changes in KCC2 and the development of chronic pain/spasticity. Injury causes a reduction in KCC2 sufficient to transform the action of GABA within 24 h [224]. Yet, spasticity and enhanced pain generally do not develop until weeks later [239,339,427]. Again, the findings imply that a downregulation in KCC2 is not sufficient to produce these effects—that other processes and events play an essential role. Key processes may include the engagement of nociceptive fibers, hemodynamic dysregulation, and factors related to stress.

Additional research is also needed to explore the contribution of these processes to other pathophysiological features of SCI. One unknown concerns the contribution of ionic plasticity to autonomic dysreflexia. Likewise, while it is known that a downregulation of KCC2 contributes to a maladaptive consequence of morphine treatment (spinally mediated hyperalgesia) [333], it is not known whether this effect mediates the adverse effect acute morphine has on tissue sparing and long-term recovery [428,429]. Finally, it should be noted that our review has focused upon how serotonergic fibers and ionic plasticity affect lumbosacral function. A parallel line of work has explored the consequences of cervical injury on respiratory function, demonstrating a BDNF-dependent benefit of intermittent hypoxia [9,430]. Here too, descending serotonergic fibers play an essential modulatory role. However, in this model, ionic plasticity may contribute little to pathophysiology or recovery [431].

## Figures and Tables

**Table 1:** Distribution and function of alternative 5-HT receptors (SC = spinal cord; SCI = spinal cord injury; DRG = dorsal root ganglion).

Receptor		Receptor Type	Location in SC	Normal Function	Function after SCI
5-HT_1_	1A	Gi/o	Primarily in laminae I and II [35]; Cell bodies in dorsal and ventral horns and intermediate zone [36,37,38]	Antinociception [39,40]; Pronociception [41,42]; Enhances motoneurons [43]; Micturition reflex facilitation [44,45,46,47,48,49]; Inhibits motor function [50,51]	Locomotor recovery [52,53,54]; Antinociception [39]
	1B		Intermediate zone [35,55]; Dorsal horn (laminae I and IV) [35,55,56]	Antinociception [40,57]	Mitigating spasms [58]; Inhibits mono- and polysynaptic reflexes [58,59]
	1D		Superficial dorsal horn [60,61]; γ motoneurons in ventral horn [62]	Antinociception [57]; Inhibits monosynaptic reflexes [63]	Inhibits bladder activity [64]; Inhibits mono- and polysynaptic reflexes [65]
	1E				
	1F		DRG [66]	Antinociception [67,68]	Mitigating Spasms [58]
5-HT_2_	2A	Gαq	Laminae II and II of dorsal horn [69]; Ventral horn [70]	Antinociception [71,72]; Pronociception [40,73,74]; Protects adaptive learning [75]; Sexual behavior [76,77]; Micturition reflex facilitation [78,79]; Motor function [50,51,80,81,82]	Functional motor recovery [83,84]; Respiratory recovery [85]; Bladder recovery [79]
	2B		Dorsal horn [86]; DRG [86,87]; Motoneurons [88]	Pronociception [86,87,89]	Functional motor recovery [83]; Mitigates spasms [83,90]; Respiration [88]
	2C		Most parts of spinal gray (except lamina II) [91] and superficial dorsal horn [92]	Spinal reflexes [93]; Inhibit motor activity [94]; Micturition reflex inhibition [78,79,95,96]	Functional motor recovery [83,97]; Mitigates spasms [83,90]
5-HT_3_	3A	Ligand-gated ion channel	In spinal gray matter [91,98]; Laminae VI through X in dorsal horn [91]; DRG [99]	Pronociception [40,100,101]; Antinociception [102,103]; Micturition facilitation [104]	Motor recovery [105]
	3B				
	3C				
	3D				
	3E				
5-HT_4_		GαS	Ventral horn [106]	Pronociception [107]; Micturition reflex facilitation [45]	Locomotor recovery [54,108,109]
5-HT_5_	5A	Gi/o	Laminae I and II of dorsal horn [110]	Antinociception [111,112]; Micturition function [110]	
	5B		Not expressed in humans [113]		
5-HT_6_		GαS	Superficial dorsal horn and lamina IX [114]; DRG [115]	Pronociception [116]	
5-HT_7_		GαS	Superficial laminae [117]; Laminae VII and VIII [118]	Pronociception [119,120]; Antinociception [120,121]; Micturition reflex facilitation [122,123]; Motor function [51,81,82,124]	

**Table 2:** Common serotonergic agonists and antagonists.

Receptor		Agonists	Antagonists	Non-Selective Agonists	Non-Selective Antagonists
5-HT_1_	1A	8-OH-DPAT (5-HT1A/7) [125]; Diprpyl-5-CT and Gepirone [126]	WAY-100635 [127]; BMY 7378, NAN-190, MDL 75005 EF; SDZ 216525 [126]; NAD-299 [49]		Propranolol [116]; Spiperone and Pindolol [126]
	1B	TFMPP and mCPP [128]; L-694247, RU 24969 [129], 5-CT, CP 93129 [126]	Quipazine [128], Methiothepin, SB-244289 and SB-216641 [126]		
	1D	Gr-46611 [130]	BRL-15572 [126,130]; Ketanserin and Ritanserin [126]		
	1E				
	1F	Lasmiditan (COL-144; LY573144) [68]; LY344864 and LY334370 [126]			
5-HT_2_	2A	DOI (5-HT_2A/2C_) [131]; TCB-2 [71]; Quipazine [132]	Ketanserin; Ritanserin (5-HT_2A/2c_) [133]; MDL 100907, SB 200646A, SB 206553 [126]	DOM [134]; SB 200646 (5-HT_2B/2C_) and SB 206553 (5-HT_2B/2C_) [126]	Ketanserin [116]; Methysergide (5-HT_1/2_) [135]
	2B	α-methey-5-HT [136]; SB 204741, Yohimbine [126]	RS-127445 [95]; SB 204741 [126]		
	2C	MK-212 [130]; WAY-161503 [71]; RO-600175 [96]	D-MC-5-H-dibenzo [130]; N-desmethylclozapine [71]; SB-242084 [96,126], RS-102221 [126]		
5-HT_3_	3A	SR-57227 [137]; 2-methyl-5-HT [134]; PBG [126]	Ondansetron (Zofran3), Alosetron [138], Granisetron, Tropisetron, MDL 72 222 [126]		Tropisetron [116]
	3B				
	3C				
	3D				
	3E				
5-HT_4_			GR 113808 and SB204070 [126]		
5-HT_5_	5A				
	5B				
5-HT_6_		EMD-386088 [117]	SB-271046 [139]; SB-399885, SB-258585 [116]; Ro 04-6790 and Ro 63-0563 [126]		
5-H7_1_		LP-211 [140]; E-57431, AS-19 [141]; E-55888 [142]	SB-269970 [119,143]; SB-656104 [140]; SB-258719 [141,142]; LP44 [123]		

**Table 3:** Distribution and function of alternative norepinephrine receptors (SC = spinal cord; SCI = spinal cord injury).

Receptor		Receptor Type	Location in SC	Normal Function	Function after SCI
α_1_	α_1A_	G_q/11_	Dorsal horn, intermediate cell column, and ventral horn [17]; motoneurons [356]	Antinociception [17]; motor behavior, pronociception, autonomic processing [17]	Spontaneous motoneuron activity7; spasticity [357]; Sympathetic neurovascular function [358,359]; micturition [360,361]
	α_1B_				
	α_1C_				
α_2_	α_2A_	G_i/o_	Superficial dorsal horn and deeper laminae, and lamina X [17]; motoneurons [356]	Antinociception [17,362,363]; inhibits sympathetic outflow [17]	Locomotor recovery [364]; mediates bowel dysfunction [365]; reflex/muscle spasticity [366,367]; neurological recovery [368]
	α_2B_		Dorsal horn [17]		
	α_2C_		Dorsal horn and DRG [17]; motoneurons [356]		
β	β_1_	Gs		Cardiac function [369]	Micturition [370], locomotor recovery [371,372], cardiac function [369]
	β_2_				
	β_3_				
	β_4_				

**Table 4:** Common noradrenergic agonists and antagonists.

Receptor		Agonists	Antagonists	Non-Selective Agonists	Non-Selective Antagonists
α_1_	α_1A_	Methoxamine (A61603) [357]	WB4010 [357], prozosin [357], BRL44408 [373], silodosin, naftopidil [374], tamsulosin [361]	REC15/2739 [357]; methoxamine [358], phenylephrine [358,359,360]	Terazosin [360,375]
	α_1B_				
	α_1C_				
α_2_	α_2A_	Clonidine, UK14303 [357,376], Guanfacine [377]	Atipamezole [373]	Dexmedetomidine [368,378], guanabenz, UK-14304 [376], tianidine [367]; medetomidine [379]	Yohimbine, RX821001(2) [357], rauwolscine, idazoxan [376], efaroxan [373]
	α_2B_		ARC239 [373]		
	α_2C_				
β	β_1_	Dobutamine [369]			Propranolol [380,381], carvedilol [382,383], nadolol [384]
	β_2_	Formoterol [371,372]	ICI118551 [385]		
	β_3_	Vibegron [370]	SR59230A [385]		
	β_4_				

**Table 5:** Distribution and function of alternative dopamine receptors (SC = spinal cord; SCI = spinal cord injury).

Receptor		Receptor Type	Location in SC	Normal Function	Function after SCI
D_1_-like	D_1_	G_q_	Throughout the spinal cord [17]	Pronociception [17]	Micturition [398,399], cardiovascular function [400], pronociception [401]
	D_5_				
D_2_-like	D_2_	G_i/o_	Superficial laminae and lamina X [17]	Antinociception, pronociception [17]	Micturition [398,399], cardiovascular function [400],
	D_3_		Dorsal horn [17]		antinociception [401]
	D_4_		Dorsal horn (check this one) [17]		

**Table 6:** Common dopaminergic agonists and antagonists.

Receptor		Agonists	Antagonists	Non-Selective Agonists	Non-Selective Antagonists
D_1_-like	D_1_	SKF 38393 [398,399,402] [403]	SCH 23390 [398,399,400,402,403], SCH 39166 [401,404]	Aripiprazole [405], apomorphine [300,398,400,406], SKF 83959 [402]	
	D_5_				
	D_2_	Quinpirole [399,402,403,407], Ropinirole [408], sumanirole [409], B-HT 920, bromocriptine [410,411], LY 141865 [412]	Remoxipride [398,399], domperidone [400,413], metoclopramide [400], eticlopride [414], L-741,626 [402], (−)-sulpiride [403,410], haloperidol [411]		
D_2_-like	D_3_	Pramipexole [401,404], ropinirole [408]			
	D_4_				

## Data Availability

Not applicable.
